# Comparative Assessment of the Antimicrobial Efficacy of Photoactivated Disinfection Employing Distinct Photosensitizers: A Morphological Analysis Using Scanning Electron Microscopy and Confocal Laser Scanning Microscopy

**DOI:** 10.7759/cureus.71008

**Published:** 2024-10-07

**Authors:** Yash Sinha, Akansha Tilokani, Prasanti Pradhan, Gaurav Patri, Aditi Gupta

**Affiliations:** 1 Department of Conservative Dentistry and Endodontics, Kalinga Institute of Dental Sciences, Bhubaneswar, IND; 2 Department of Pedodontics and Preventive Dentistry, Kalinga Institute of Dental Sciences, Bhubaneswar, IND

**Keywords:** chlorin e6, clsm, irrigation, lasers, photoactivated disinfection, sem

## Abstract

Background

The complex morphology of the root canal system and bacterial infiltration to greater depths precludes complete debridement by mechanical preparation alone. Despite promising characteristics of photodynamic inactivation (PDI) and different photosensitizers (PSs), there has been limited research on the antibacterial efficacy of chitosan (CS)-based PS combinations for root canal disinfection. We aimed to evaluate and compare the antimicrobial efficacy of photoactivated disinfection (PAD) using two different PSs as an adjunct to final irrigation in root canal treatment.

Methodology

Forty single-rooted human teeth were divided into four groups: Group A (positive control) = chemo-mechanical debridement (5.25% sodium hypochlorite (NaOCl)); Group B = chemo-mechanical debridement (5.25% NaOCl) + 15 μg/ml of methylene blue (MB) with T1 = 30 sec + diode laser (650 nm) and T2 = 30 sec; Group C = chemo-mechanical debridement (5.25% NaOCl) + 15 μg/ml of chlorin e6 (Ce6) with T1 = 30 sec + diode laser (650 nm) and T2 = 30 sec; Group D = chemo-mechanical debridement (5.25% NaOCl) + 15 μg/ml of Ce6 and CS with T1 = 30 sec + diode laser (650 nm) and T2 = 30 sec. Bacterial culture, scanning electron microscopy (SEM), and confocal laser scanning microscopy (CLSM) analysis were used to examine microbial coverage. The data were analyzed using IBM SPSS Statistics software, version 25 (IBM Corp., Armonk, NY); ANOVA followed by Tukey’s post-hoc analysis was used to compare colony counts among the four groups.

Results

While inter-group comparison of pre-irrigation bacterial counts did not reveal a significant difference, post-irrigation counts were greater for NaOCl, followed by MB, Ce6, and Ce6+CS. However, all groups showed a significant reduction in counts post irrigation (Group A = 20.28 vs. 1.36; Group B = 20.12 vs. 1.11; Group C = 20.16 vs. .62; Group D = 20.20 vs. .33; all values in *10).

Conclusion

Based on colony counts and SEM and CLSM analysis, we found better anti-microbial properties for Ce6+CS, followed by Ce6, MB, and NaOCl, despite not having a difference in their colony counts before irrigation.

## Introduction

Apical periodontitis is closely linked to microbial infections within the root canal system [1]. While polymicrobial primary endodontic infections predominantly involve Gram-negative anaerobic bacteria responsive to ecological changes induced by chemo-mechanical preparation, secondary infections, in contrast, have limited bacterial species well-adapted to survive fluctuating environments. They often exhibit antimicrobial resistance, leading to treatment failure in cases of inadequate disinfection procedures [2]. Of the numerous species coalescing to form a complex biofilm, *Enterococcus faecalis* (*E. faecalis*) and streptococci are commonly isolated from recurrent root canal infections [2, 3]. A thorough debridement by mechanical preparation alone is not possible due to the intricate morphology of the root canal system and bacterial infiltration up to 1500 µm into the root canal [4,5]. Current techniques recommend using a substantial volume of irrigating solution for effective cleaning [6].

Recent advancements in photodynamic inactivation (PDI) have led to their use for adjunctive treatment in endodontics, periodontics, and peri-implantitis. Protoporphyrin IX, which is present in some bacteria and higher organisms, is biosynthetically related to chlorin e6 (Ce6), a second-generation photosensitizer that is derived from chlorophyll a [7]. Chitosan (CS) is another natural cationic polymer with numerous modifiable sites (-OH, -NH₂) that binds effectively to negatively charged bacterial surfaces. This electrostatic interaction promotes CS's potential as a carrier for photosensitizers (PS) [8]. Despite its promising characteristics, there has been limited research on the formulation development and antibacterial efficacy of CS-based photosensitizer combinations for root canal disinfection. We aimed to evaluate and compare the antimicrobial efficacy of photoactivated disinfection (PAD) using two different photosensitizers as an adjunct to final irrigation in root canal treatment.

## Materials and methods

This study was approved by the Institutional Ethics Committee, Kalinga Institute of Medical Sciences, Kalinga Institute of Industrial Technology (KIIT) (Deemed to be University), Bhubaneswar, Odisha, under the ethical code of KIIT/KIMS/IEC/841/2022.

Sample preparation

Forty single-rooted human teeth were sectioned from the middle third of the root to obtain 6 mm segments between the cementoenamel junction and the apical third. The patency of the apical foramina was confirmed using hand files, and the working length was determined radiographically. Canals were sequentially enlarged to size X3 ProTaper, and canals were irrigated alternately with 20 ml of 5.25% sodium hypochlorite (NaOCl) and 2 ml of 17% ethylenediamine tetraacetic acid (EDTA) for three minutes using a 30G side-vented needle. The canals were then rinsed with 2 ml of saline to remove residual irrigants. The apical foramina were then sealed externally, and samples sterilized.

Preparation of Ce6 and Ce6+CS

A 0.6 mg/mL concentrated Ce6 solution was prepared using Magar et al.’s method [9]. Similarly, CS solution was prepared at a concentration of 1.7 mg/mL. They were mixed through sonication at 37°C for 30 minutes to form a Ce6+CS complex.

Inoculation of *E. faecalis* and pre-irrigation sample collection

Trypticase soy broth (TSB) was used to subculture standard strains of *E. faecalis* (ATCC29212) under 37°C for 24 hours. The resulting turbid suspension was used to inoculate the root canals. Using a sterile micropipette, 15 μl of this suspension was added to each root canal. The teeth were then placed in Eppendorf tubes and incubated at 37°C in a humid environment for 21 days. To preserve bacterial viability, root canals were refilled with *E. faecalis* inoculum every three days throughout this incubation period. We divided the teeth into four groups: Group A (positive control) = chemo-mechanical debridement (5.25% NaOCl); Group B = chemo-mechanical debridement (5.25 NaOCl) + 15 μg/ml of methylene blue (MB) with T1 = 30 sec + diode laser (650 nm) and T2 = 30 sec; Group C = chemo-mechanical debridement (5.25% NaOCl) + 15 μg/ml of Ce6 with T1 = 30 sec + diode laser (650 nm) and T2 = 30 sec; Group D = chemo-mechanical debridement (5.25% NaOCl) + 15 μg/ml of Ce6 and CS with T1 = 30 sec + diode laser (650 nm) and T2 = 30 sec

Chemo-mechanical debridement

Using a ProTaper® F2 file (Ø = 0.25 mm, Dentsply Sirona, Charlotte, NC)., mechanical preparation was done, and the canals were irrigated with 5.25% NaOCl for one minute. Sterile saline was used for final irrigation. To neutralize any remaining NaOCl, the canals were flushed with 5% sodium thiosulfate for one minute.

Photoactivated disinfection

Root canals in photodynamic therapy (PDT) groups (Groups B, C, and D) were filled with approximately 15 µL of 0.05 mM MB, 0.05 mM Ce6, and 0.05 mM Ce6+CS, respectively, using a 30G side-vented needle and agitated with a K-file #15. The solutions were left for 30 seconds (T1) before laser application. Using the following parameters, a 650 nm diode laser was employed for PAD: The continuous mode power was 100 mW, and the optical fiber diameter was 400 μm. A rubber stopper was placed on the laser fiber to keep the working length at 1 mm. Laser irradiation for 30 seconds (T2) was performed with helical movement from the apex to the cervical portion of each canal.

A gates glidden drill (Mani®, Utsunoniya, Tochigi, Japan) size no. 3, 1.1 mm in diameter, was used to gather dentinal shavings in one stroke before and after the chemo-mechanical debridement and laser activation.

One ml of sterile TSB was placed in a microcentrifuge tube along with the collected dentinal shavings. Using a sterile microtip, 100 µl of dentinal shavings-containing broth was taken and then transferred to a different tube that held 900 µl of sterile TSB. Then, each tube's contents were successively diluted from 10-1 to 10-4. Using an L-shaped glass rod, 300 µl of diluted dentinal shavings were streaked evenly and tripled. These TSB plates were incubated for 24 hours at 37 degrees Celsius. Following the incubation period, the colonies were counted and the readings tabulated. The remaining viable microbial population was determined by calculating the total number of colony-forming units (CFUs) [10].

Scanning electron microscopy (SEM) analysis

After treatment, one sample from each group was selected, and SEM images of the canal walls were examined to assess microbial coverage. A four-score scale (covering less than 5%, 5%-33%, 34%-66%, and 67%-100% of the dentine) was used to quantify microbial coverage [11].

Confocal laser scanning microscopy (CLSM) analysis

The viability profile of Ce6 and Ce6+CS was assessed to determine their efficacy as photosensitizers. The proportion of live and dead bacteria was determined using fluorescent staining followed by imaging, as per Daood et al.'s protocol [12].

Statistical analysis

The data were analyzed using IBM SPSS Statistics software, version 25 (IBM Corp., Armonk, NY). Colony counts before and after irrigation were presented as mean ± standard deviation; before and after values were individually compared among the four groups using ANOVA followed by Tukey’s posthoc analysis. Before vs. after comparison of values in each group was done using a paired t-test. A p-value of ≤.05 was considered significant for all analyses.

## Results

Antimicrobial assessment

While inter-group comparison of pre-irrigation bacterial counts did not reveal a significant difference, post-irrigation counts were greater for NaOCl, followed by MB, Ce6, and Ce6+CS (Tables 1, 2). 

**Table 1:** Inter-group comparison of colony counts (before irrigation in CFU/ml) among the four groups using ANOVA NaOCI: sodium hypochlorite; CFU: colony-forming units

Group	Mean (*10^2^)	Standard deviation	Minimum	Maximum	p-value
5.25% NaOCl	20.28	.713	19.00	21.20	.938
Methylene blue	20.12	.509	19.60	21.00	
Chlorin e6	20.16	.497	19.60	21.00	
Chlorin e6 with chitosan	20.20	.596	19.60	21.00	

**Table 2:** Inter-group comparison of colony counts (after irrigation in CFU/ml) among the four groups using ANOVA NaOCI: sodium hypochlorite; CFU: colony-forming units

Group	Mean (*10^2^)	Standard deviation	Minimum	Maximum	p-value
5.25% NaOCl	1.36	.051	1.24	1.42	< .001
Methylene blue	1.11	.063	1.04	1.24	
Chlorin e6	.62	.085	.54	.80	
Chlorin e6 with chitosan	.33	.157	.24	.76	

However, all groups showed a significant reduction in counts post irrigation (Tables 3-4).

**Table 3:** Post hoc comparison for after irrigation colony counts NaOCI: sodium hypochlorite

Group 1	Group 2	Mean difference	p-value
5.25% NaOCl	Methylene blue	.250	< .001
	Chlorin e6	.738	< .001
	Chlorin e6 with chitosan	1.03	< .001
Methylene blue	Chlorin e6	.488	< .001
	Chlorin e6 with chitosan	.782	< .001
Chlorin e6	Chlorin e6 with chitosan	.294	< .001

**Table 4:** Before-after comparison of colony counts in each of the four groups using a paired t-test NaOCI: sodium hypochlorite

Group	Before (*10^2^)	After (*10^2^)	p-value
5.25% NaOCl	20.28	1.36	< .001
Methylene blue	20.12	1.11	< .001
Chlorin e6	20.16	.62	< .001
Chlorin e6 with chitosan	20.20	.33	< .001

Scanning electron microscopy analysis

**Figure 1:**
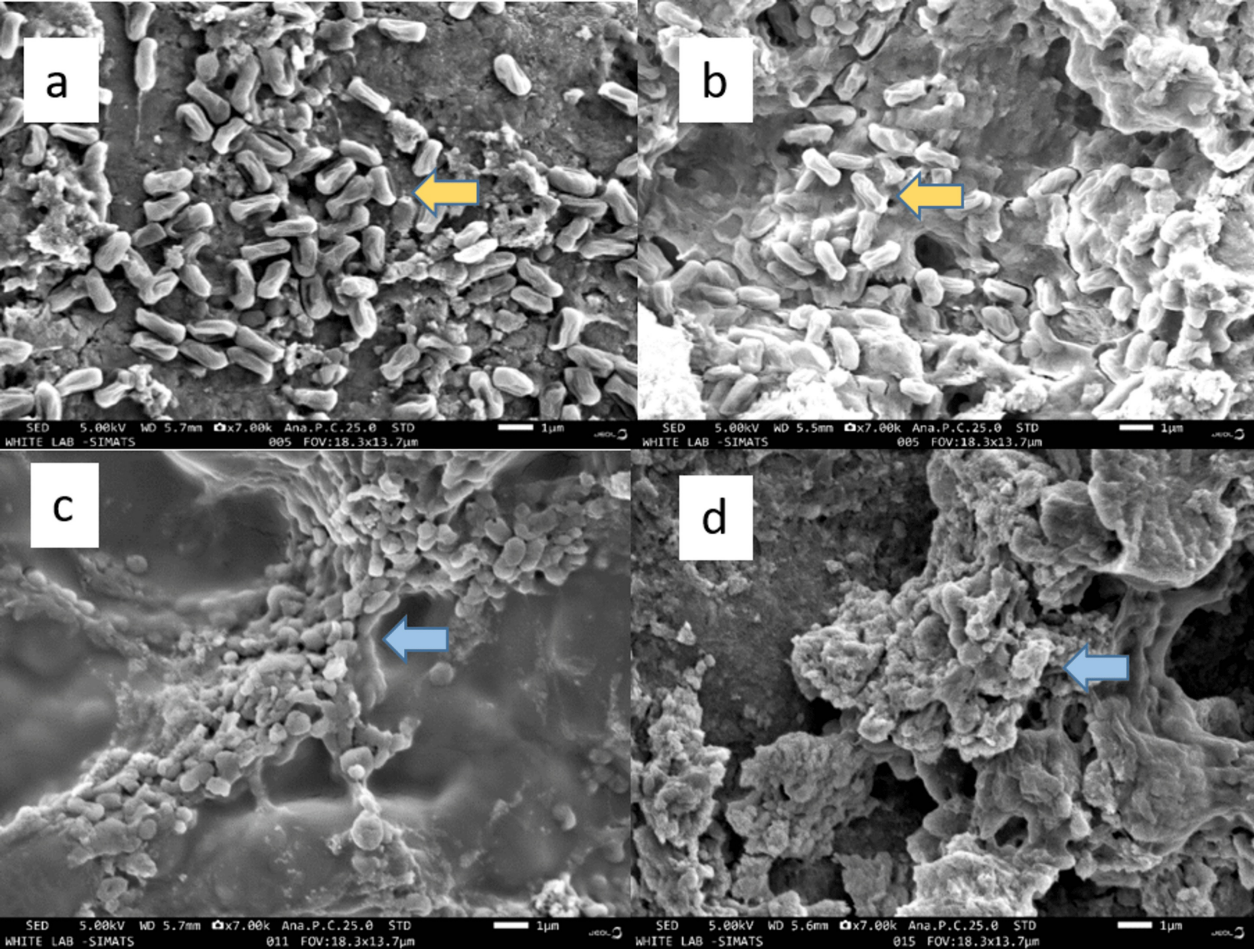
a: chemo-mechanical debridement (5.25% NaOCl); b: chemo-mechanical debridement (5.25% NaOCl) + PAD using methylene blue; c: chemo-mechanical debridement (5.25% NaOCl) + PAD using chlorin e6; d: chemo-mechanical debridement (5.25% NaOCl) + PAD using chlorin e6 with chitosan The yellow arrow in images A and B indicates intact bacteria. The blue arrow in images C and D indicates an irregular extracellular matrix within dentinal tubules. NaOCI: sodium hypochlorite; PAD: photoactivated disinfection

The SEM images verified the presence of thick biofilm of residual *E. faecalis* bacteria on root canal dentin of Groups A and B with a coverage of 34%-66% (Figures 1a-1b), followed by Group C and Group D with a coverage of 5%-33% and less than 5%, respectively (Figures 1c-1d).

Confocal laser scanning microscopy analysis 

**Figure 2:**
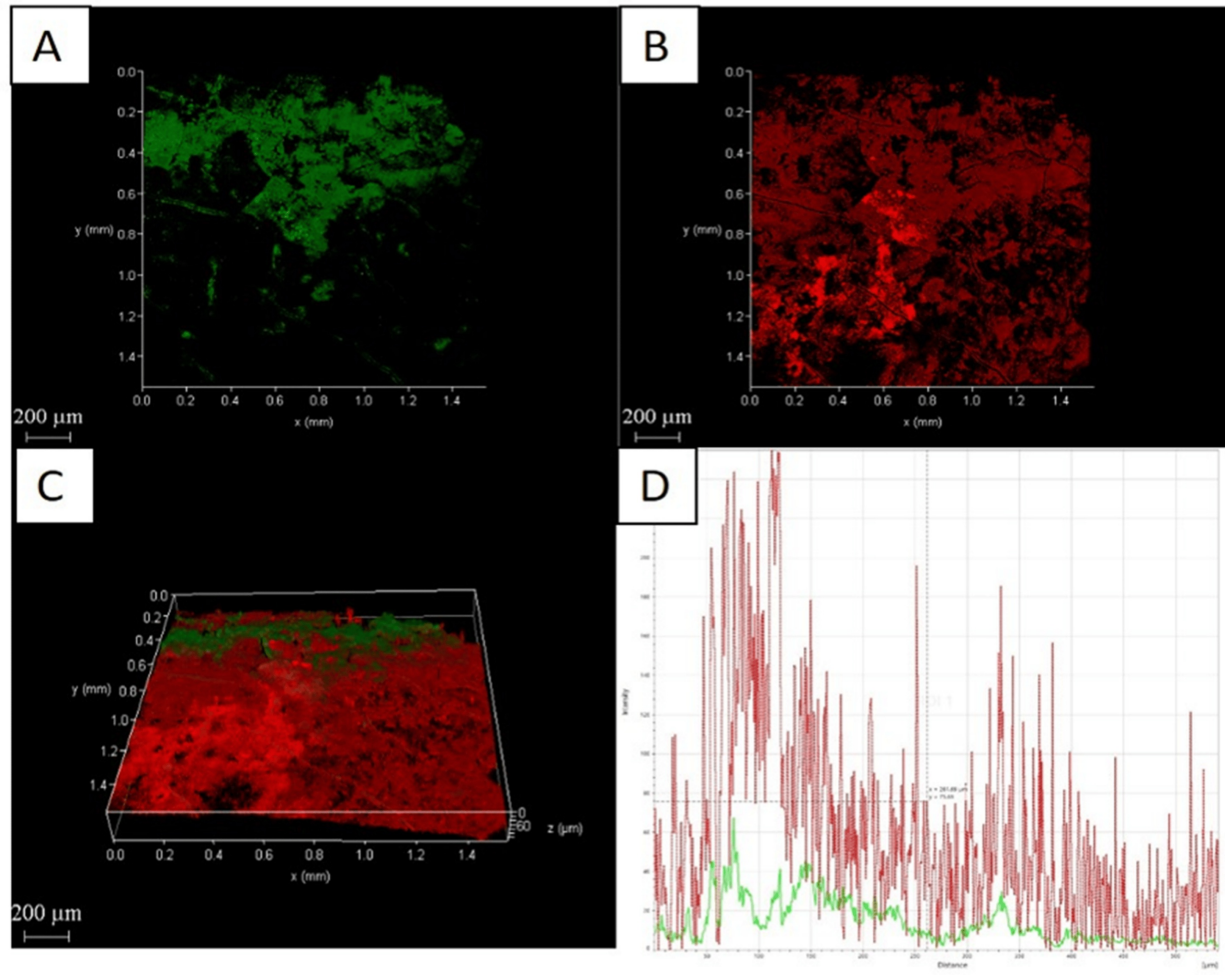
Chemo-mechanical debridement (5.25% NaOCl) A: 2D image of live bacteria; B: 2D image of dead bacteria; C: 3D image of live and dead bacteria; D: graphical representation of live and dead bacteria NaOCI: sodium hypochlorite

**Figure 3:**
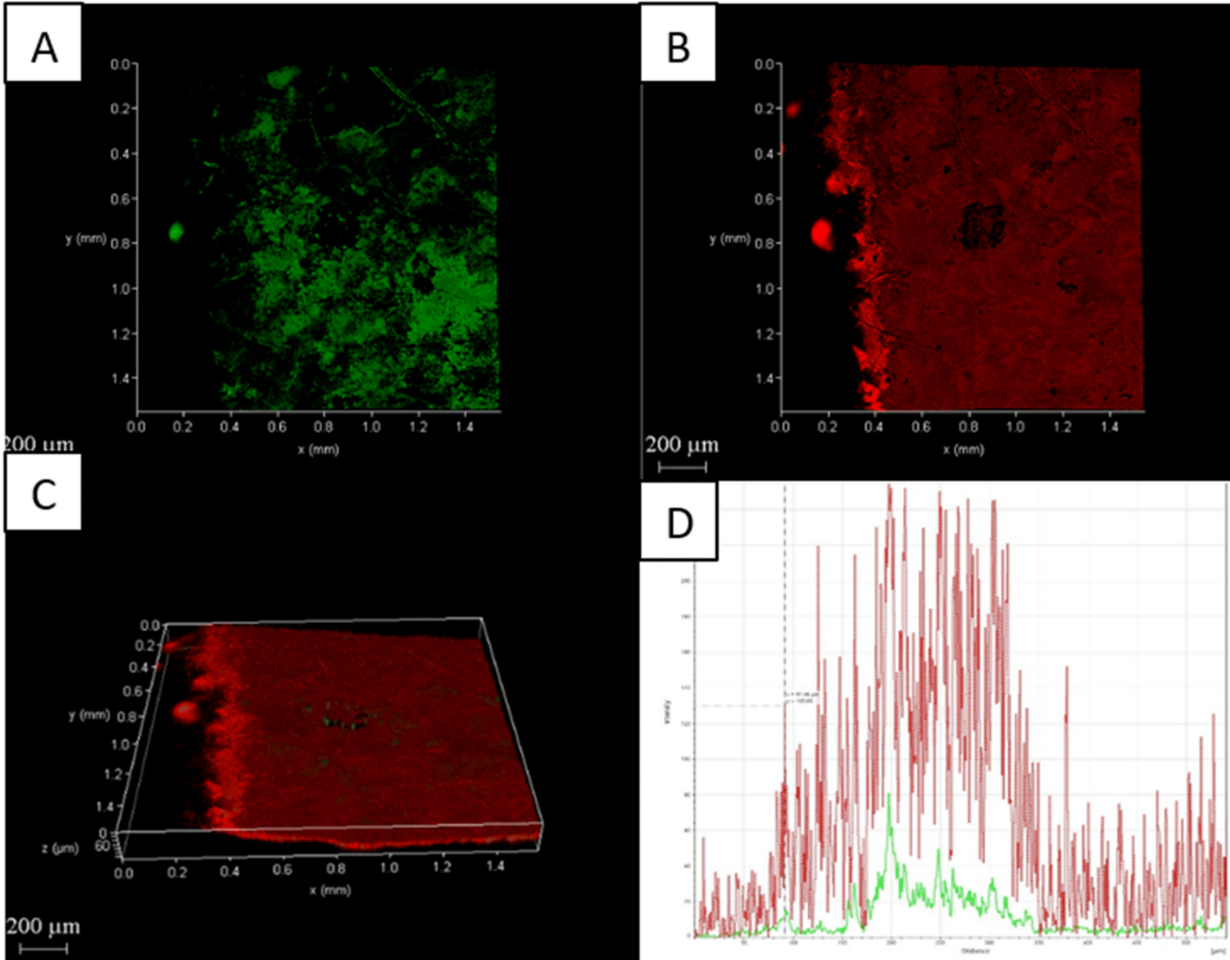
Chemo-mechanical debridement (5.25% NaOCl) + PAD using methylene blue A: 2D image of live bacteria; B: 2D image of dead bacteria; C: 3D image of live and dead bacteria; D: graphical representation of live and dead bacteria NaOCI: sodium hypochlorite; PAD: photoactivated disinfection

**Figure 4:**
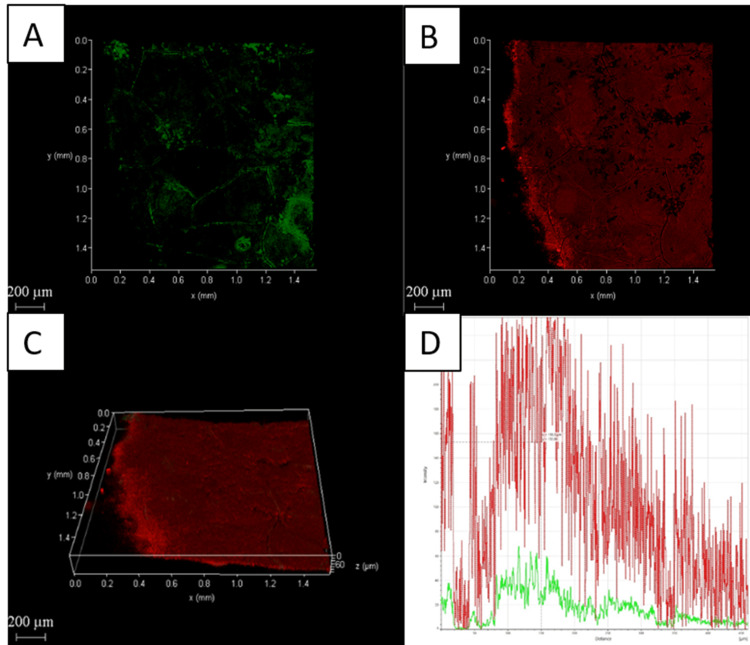
Chemo-mechanical debridement (5.25% NaOCl) + PAD using chlorin e6 A: 2D image of live bacteria; B: 2D image of dead bacteria; C: 3D image of live and dead bacteria; D: graphical representation of live and dead bacteria NaOCI: sodium hypochlorite; PAD: photoactivated disinfection

**Figure 5:**
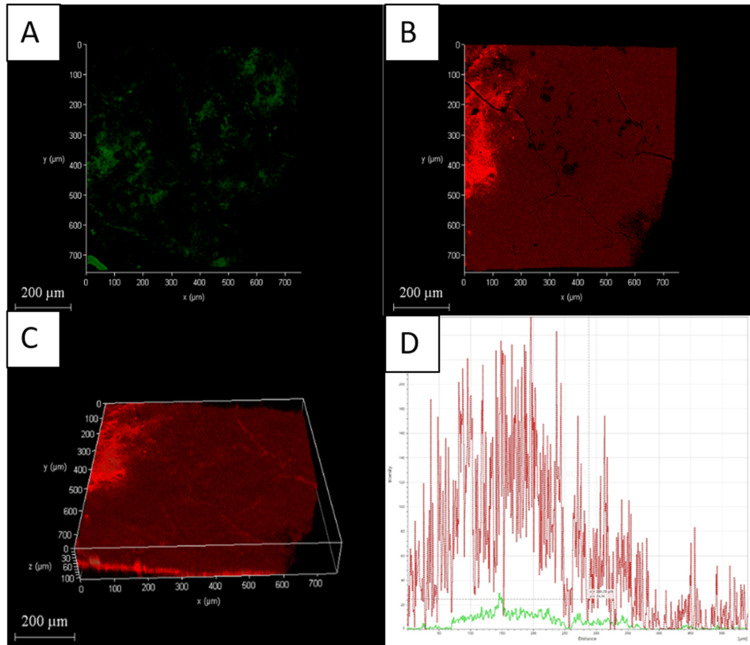
Chemo-mechanical debridement (5.25% NaOCl) + PAD using chlorin e6 with chitosan A: 2D image of live bacteria; B: 2D image of dead bacteria; C: 3D image of live and dead bacteria; D: graphical representation of live and dead bacteria NaOCI: sodium hypochlorite; PAD: photoactivated disinfection

The CLSM images also showed the amount of dead cells in dentin was highest with Group D compared to the other three groups (Figures 2-5).

## Discussion

Successful endodontic therapy relies on comprehensive chemo-mechanical disinfection, to disrupt the microbial biofilm [13]. We used a mono-species *E. faecalis* biofilm. Compared to younger biofilms, mature *E. faecalis* in root canals for 21 days shows increased resistance to disinfecting solutions [14, 15]. Therefore, the efficacy of the photosensitizer as an adjunct to final irrigation was evaluated specifically against these 21-day mature biofilms.

In order to ensure consistent inoculation of photosensitizer and bacteria, mid-root dentin blocks were standardized to a height of 6.0 mm within the root canal. It was observed that* E. faecalis* rapidly invaded the dentinal tubules, with the infection front reaching up to 1000 µm in some blocks. Consequently, the samples were analyzed at depths of 500-600 µm within the dentinal tubules to assess the extent of microbial invasion [16].

Photodynamic therapy uses any one of halogen lamps, light-emitting diodes (LEDs), and lasers [17]; diodes are more affordable and portable. Phenothiazines, or synthetic nonporphyrin compounds, are commonly used dyes [18,19].

We compared the efficacy of Ce6 and Ce6+CS against MB; Ce6 is more suitable for endodontic applications due to lower toxicity to pulp and periapical tissues [20]. Although Nie et al. [21] found both Ce6 and MB-based photodynamic inactivation effective in managing biofilms, Ce6 was a better photosensitizer at concentrations below 200 μM [21]. Chitosan nanoparticles (CSNPs) enhance the effectiveness of antimicrobial photodynamic therapy (aPDI) [22]. *Streptococcus mutans* on bovine dentine blocks showed a reduction of approximately 1 log when treated with a diode laser in conjunction with chloro-aluminum phthalocyanine encapsulated in CSNPs [23].

Pulp stones (PSs) encapsulated in polymeric nanoparticles, such as CSNPs, offer advantages over free-form PSs dissolved only in organic or physiological media, like increased selectivity for target cells and the need for lower PS concentrations to generate reactive oxygen species effectively [24]. In another study, a combination of CS and photoditazine (a Ce6-based dye) successfully reduced *Streptococcus mutans *in both planktonic and biofilm forms [25].

The potential for tooth discoloration and staining following MB application related to its concentration and the duration of pre-radiation exposure is concerning [26]; MB is commonly employed at concentrations of 15 to 400 µg/mL [27, 28] and in vivo concentrations between 50 µg/mL and 10 mg/mL [29]. We used a solution of CS and Ce6 at concentrations of 0.6 mg/ml and 1.7 mg/ml.

The pre-radiation period in PDT is crucial, as the photosystem, after penetrating the dentinal tubules, demonstrates a profound antimicrobial effect following irradiation [26]. Lasers with wavelengths between 600 and 805 nm have output powers ranging from 40 mW to 5 W and irradiation times between 0.5 and 10 min [30]. For our study, pre-irradiation and irradiation times of 30 seconds were maintained following Tenore et al.'s methodology [31].

The spatial distribution and viability of the bacteria, with the exception of dentinal tubules, could not be analyzed using quantitative analysis of bacteria to determine a log reduction in CFUs in infected dentine before and after PAD [32]. In the present study, the after-irrigation bacterial counts were significantly lower in the Ce6 + CS group, followed by the Ce6 group. In addition, the difference in count from pre-to-post was also highest for the Ce6 + CS group. This is similar to a study by de Souza et al. [25] that found a reduction of 4.65 log10 on using Ce6 with chitosan as compared to Ce6 alone.

Our findings are also in line with those of Nie M et al. [21], in finding better antimicrobial activity for Ce6 than MB, thus supporting that PDI can be considered efficient against *Streptococcus mutans* biofilm control and highlighting that Ce6-mediated PDI has excellent clinical potential in the treatment of dental biofilm. Another study by Diogo et al. [20] too demonstrated better antimicrobial efficacy for Ce6 as compared to NaOCl against mixed biofilms of *E. faecalis* with *Candida albicans*.

Leticia et al. [33] compared the antibacterial effects of PDT with MB at 15 µg/mL against chemo-mechanical debridement using NaOCl for* E. faecalis*. In contrast to our study, they could not show a significant supplemental effect on instrumentation and irrigation procedures concerning intracanal disinfection for PDT with MB.

To ascertain PDT's antibacterial effects on *E. faecalis*, we employed bacterial culture, CLSM, and SEM. Scanning electron microscopy directly observes *E. faecalis* distribution and bacterial morphology on the sample surface [10]. After irrigation, chemo-chemical debridement with NaOCl and PAD using Ce6 and CS showed the least *E. faecalis* coverage of less than 5% on the root canal dentin. Though the biofilm and bacteria on the dentin were completely removed, there existed some irregular “extracellular matrix” within dentin tubules (Figure 1d). Next followed Group C with an *E. faecalis* coverage of 5%-33% of dentin. Most bacteria were degraded and deformed, but some dispersed inside the dentinal tubule (Figure 1c). Groups A and B showed the highest *E. faecalis* coverage of 34%-66%, with a significant number of bacteria intact (Figures 1a-1b). A few “bacteria corpses” were scattered, and their spherical shape crumpled.

The distribution of dead bacteria in the biofilm is clearly visible when fluorescence staining and CLSM are used together. This method is highly sensitive and easily replicable [34]. Group D had a higher percentage of dead bacteria on the biofilm surface of their root canals than the other three groups, as shown in Figures 2-5.

Our study was limited in using a single-species biofilm model of *E. faecalis*, despite endodontic microflora being polymicrobial; second, our biofilm model containing *E. faecalis* used on extracted teeth did not completely replicate the oral environmental conditions. We recommend future research using a multispecies biofilm model, with emphasis on the in vitro and in vivo efficacy of these intracanal medications.

## Conclusions

Based on colony counts and SEM and CLSM analysis, we found better anti-microbial properties for Ce6+CS, followed by Ce6, MB, and NaOCl, despite not having a difference in their colony counts before irrigation. The antimicrobial activity of chlorin e6 and its combination with various other nanoparticles has not been studied in depth; therefore, further research is required to understand and elucidate its mechanism of action, especially at the cellular level. However, based on the results from this study, it can be stated that Ce6 when combined with a nanoparticle like CS can be proposed as a potential photosensitizer to be used in the future.
